# Livelihood changes matter for the sustainability of ecological restoration: A case analysis of the Grain for Green Program in China's largest Giant Panda Reserve

**DOI:** 10.1002/ece3.3844

**Published:** 2018-03-14

**Authors:** Jianying Xu, Qing Wang, Ming Kong

**Affiliations:** ^1^ College of Resource, Environment and Tourism Capital Normal University Beijing China

**Keywords:** cropland conversion, ecological compensation, Grain for Green Program, livelihood changes, payment for ecosystem services

## Abstract

Payments for ecosystem services (PES) are expected to promote ecological restoration while simultaneously improving human livelihoods. As an adaptive management tool, PES programs should be dynamic and adjusted according to changing natural and socio‐economic contexts. Taking the implementation of China's famous ecological restoration policy known as the Grain for Green Program (GGP) in the Wolong National Nature Reserve as an example, we analyzed changes in the livelihood capitals and strategies of local households that had participated in the GGP over a 10‐year period and discussed the implications of these changes for the next stage of the program's implementation. Data were collected from a locally implemented questionnaire in both 2004 and 2015. We found that local livelihood capitals and strategies had experienced dramatic change over the 10‐year period. Natural capital decreased and was unequally distributed among local respondents. In terms of financial capital, despite that agricultural and nonagricultural income increased, compensation from the GGP decreased and did not keep pace with increasing cost of cropland, household income and more broadly national economic development and inflation. Regarding human capital, the local labor force is facing huge transformational pressures. In particular, there is a increase in the supply of local labor force aged between 21 and 40 and the implications of this for the future of the GGP should be given more attention. The findings have demonstrated that: Some changes in participants’ livelihood were expected by the GGP but were not evenly distributed among the participants; and PES programs are embedded in changing and multi‐dimensional socio‐economic contexts, and so their design and implementation must be coordinated with other related policies if they are to achieve long‐term success.

## 1 INTRODUCTION

In terms of its scale, China's Grain for Green Program (GGP) (also known as the Sloping Land Conversion Program) is the largest payment‐for‐ecosystem‐service (PES) program in the world. By converting steep (i.e., >25°) cropland or wasteland to grassland or forest, the GGP aimed to mitigate ecological degradation, improve the provision of ecosystem services, and simultaneously change the economic structure in mountainous areas by increasing local household income and making local households’ land use and agricultural production patterns more sustainable (Li, Feldman, Li, & Daily, 2011). Launched in 1999, by the end of 2013, the GGP had resulted in 29.8 million hectares of cropland throughout China being converted to forest (State Forestry Agency, 2014). In 2014, following the completion of two stages of the program, the Chinese Central Government decided to extend the GGP to its next stage. In the next stage, GGP will be extended to another 8 years for the land converted into forest. Different from the first two stages, it is participants themselves not the government who decide whether participant GGP or not. Thus, participants’ willingness, livelihood conditions, and alternative choice, etc. will be new focus in the next stage of GGP.

Many studies have focused on the ecological and socio‐economic outcomes of the GGP. A number of these studies have found that the program has led to environmental improvements including an increased provision of ecosystem services (Cao, Chen, & Yu, 2009; Chen, Lupi, He, Ouyang, & Liu, 2009; Chen, Zhang, Zhang, & Wan, 2009; Lu, Fu, et al., 2012; Xin, Xu, & Zheng, 2008; Zhang, Zhang, Zhao, Rustomji, & Hairsine, 2008). At the same time, household livelihood changes associated with the implementation of the GGP have also received increasing attention from scholars (Liu, Li, Ouyang, Tam, & Chen, 2008; Tang, Bennett, Xu, & Li, 2013; Zhen, Fu, Lu, & Zheng, 2014). Besides the expected land‐use and income changes for farmers (Li, Yao, Yin, & Liu, 2015; Liu, Lu, & Yin, 2010; Zhen et al., 2014), there have also been other socio‐economic changes associated with earnings differentiation (Li et al., 2015), the nonfarm labor market (Kelly & Huo, 2013), and even income inequity (Li et al., 2011).

Payments for ecosystem services programs are implemented under varying and dynamic environmental, socio‐economic, political, and temporal contexts (Jacket, Kousky, & Sims, 2008). It is critical to take into account the complexities and variety of contextual and institutional settings in which a PES program operates (Muradian, Corbera, Pascual, Kosoy, & May, 2010). Therefore, substantial changes in household livelihood associated with participating in a PES program have significant implications for the long‐term success of these programs (Bremer, Farley, Lopez‐Carr, & Romero, 2014; Locatelli, Rojas, & Salinas, 2008). It is argued that PES designers should take participants’ livelihood changes into consideration so as to improve the adaptive capacity and performance of a PES program (Armitage, 2005; Mahdi & Shivakoti, 2009). Also, PES programs that result in beneficial local livelihood changes can help inform the scientific understanding of ecosystem service management and develop methodological tools for decision support (Lu, Liu, & Fu, 2012).

Consideration of contextual changes is necessary when designing and adapting a PES program. Such changes may or may not be induced by a program itself, and they can advance or impede its further implementation. Our aim in this study was to analyze the livelihood changes of participants in the GGP and discuss the implications of these changes for the next stage of the program. Our research objectives were to analyze the quantity and distribution of the changes to the participants’ livelihood capitals and strategies; explore the reasons for and significance of these changes; and identify how these changes could inform the design and implementation of the next stage of the GGP.

## 2 MATERIALS AND METHODS

### 2.1 Description of the study area

The research was conducted in the Wolong National Nature Reserve which is located in Wenchuan County, Sichuan Province, southwest China (102°2′to 102°24′ E, 30°45 to 31°25′N). The reserve has an area of 2,000 km^2^ and is famous for supporting the conservation of Giant Pandas. It is situated in the transition zone between the Chengdu Plain and the Qinghai‐Tibet Plateau. The reserve's diverse environment provides habitat for not only the Giant Panda, but also 57 other endangered animals including the Golden Monkey (*Rhinpithecus roxellanae*) and 24 species of rare plants including the dove‐tree (*Davidia involucra*).

The reserve is managed by the Administrative Bureau of the Wolong National Nature Reserve, under which there are two township governments—Wolong Township and Gengda Township. The reserve contains a total of 1,164 households and in 2013 had a population of approximately 5,954. Although Han constitute the major ethnic group in China, approximately 70% of the people living within the reserve belong to three ethnic minority groups, namely Tibetan, Chang, and Hui. Chengdu‐Xiaojin Road is the only main road connecting the local people to areas outside of the reserve. There are six administrative villages within the reserve, each with a committee responsible for management.

The GGP has been implemented in the reserve since 2000. By the end of 2003, approximately 370 ha of cropland had been converted to forest. Since then, the program has been implemented in the reserve in two stages. In the first stage, for each hectare of cropland converted to forest, the local people were compensated with 2,250 kg of grain and 300 Yuan in cash once a year for 8 years’. In the second stage, only cash compensation was provided in the amount of 3,600 Yuan per hectare of cropland converted to forest once a year for 8 years’. A third stage of the program commenced in the reserve in 2015. While researchers have devoted significant attention to the levels of enrollment and farmers’ re‐converting their previously forested land back to cropland associated with the GGP (Chen, Lupi, et al., 2009; Chen, Zhang, et al., 2009; Chen et al., 2012) and the program's sustainability in the study area (Xu, Chen, Lu, & Fu, 2007), no attention has been given to understanding the changes that have occurred from the two former stages of the GGP in the reserve and how these changes could influence the design and implementation of future stages of the program.

### 2.2 Data collection

Research data were obtained by Participatory Rural Appraisal (PRA). PRA is a conventional method for learning from farmers and is widely used to gather research information from rural people. Local livelihood capitals in 2004 and 2015 were chosen to discern changes over this 10‐year period. The 2004 livelihood capitals values were derived from a questionnaire used to evaluate the sustainability of the GGP (Xu et al., 2007). In 2015, a questionnaire was again used to collect relevant information about local livelihood capitals and local perceptions of the GGP. A total of 137 and 182 households were randomly selected for investigation in 2004 and 2015, respectively. One adult person (>18 years old) from each household was interviewed in his or her residence. To avoid potential bias, we made it clear to the participants that the survey was for academic research only and that we were not affiliated with the GGP management authority or any of the local administration authorities.

### 2.3 A sustainable livelihood analysis framework

Following previous studies (Bremer et al., 2014; Chen et al., 2013; Liang, Li, Feldman, & Daily, 2012), we used a livelihood approach as an organizing framework to better understand changes in the local socio‐economic context. The Sustainable Livelihood Framework (SLF) was used. The SLF is defined by Chambers and Conway (1992) as “the capabilities, assets (including both material and social resources) and activities required for a means of living. A livelihood is sustainable when it can cope with and recover from stresses and choices, maintain, or enhance its capabilities and assets, while not undermining the natural resource base.” The SLF encompasses five components: (1) vulnerability context; (2) livelihood capitals; (3) transforming structures and processes; (4) livelihood strategies; and (5) livelihood outcomes (DFID, 1999, pp. 1–8). These components are intended to be dynamic due to both external interventions and the activities of the rural residents. The SLF is often used in evaluating rural development projects as it helps to organize complex data into a form that summarizes and analyzes “core influences and processes” and the interactions between different factors that impact on people's livelihoods. It provides a holistic framework for understanding and exploring the dynamics of rural livelihood outcomes and strategies associated with rural development interventions.

According to the SLF, livelihoods depend on five types of capitals: natural, human, financial, material, and social. These capitals are affected by the “vulnerability context” and “transforming structures and processes.” Thus, we use livelihood capitals to characterize local livelihood changes. Livelihood strategies, which means the choices rural residents employ in the pursuit of income, security, well‐being, and other productive purposes, are used to reveal local responses to livelihood changes.

In order to relate the SLF to the GGP and our research objectives, livelihood capitals were measured at the household level and indicators were selected relating to both the capitals and the GGP to provide insight into factors that could influence the sustainability of the GGP. Indicators were selected as follows: cropland holding indicating natural capital, household income indicating financial capital, the number and educational level of household members indicating human capital. For the household material capital, the numbers of pigs and cattle owned by the household was chosen as an indicator because pig‐feeding and cattle‐feeding have a close relationship with both biodiversity conservation and agricultural practice (An, Lupi, Liu, Linderman, & Huang, 2002). Measures of natural capital, financial capital, human capital, and material capital were all included in the questionnaire in both 2004 and 2015. A measure of social capital was included in the 2015 survey but not in the 2004 survey. Despite this, we can assume that social capital has improved substantially since 2004 due to the popularity of mobiles phones and the internet. Besides livelihood capital, local livelihood strategies (i.e., employment situation of each labor adult and local perceptions of the current stage of the GGP) were also investigated in both 2004 and 2015. Due to the significance of natural and financial capital in characterizing past achievements and future implementation of the GGP, their distribution among local people were also calculated in the study. The p90/p10, which indicates economic inequality, was chosen to indicate the distribution of financial capital. The p90/p10 showed the ratio of upper bounds of the ninth decile (i.e., the 10% of people with the highest income) to that of the first decile. A Lorenz Curve, which showed the percentage of households with the smallest cropland holding, what percentage of the total cropland they have, was chosen and modified to indicate the distribution of natural capital.

## 3 RESULTS

### 3.1 Demographic variables

Table 1 outlines the respondents’ socio‐demographic characteristics. A total of 59 women (43%) and 78 men (57%) were interviewed in 2004, while 98 women (54%) and 84 men (46%) were interviewed in 2015. There were fewer respondents below 30 years of age in 2015 than in 2004 because in 2015 more young farmers were working outside the reserve. As a consequence, there were more middle‐aged respondents in 2015 than in 2004. The number of respondents with high school education increased from 15% to 28% between 2004 and 2015, while those that had only primary school education or were illiterate decreased by approximately 10%.

**Table 1:** Respondents’ socio‐demographic characteristics

Characteristics	Groups	2004			2015		
		Wolong	Gengda	Total (%)	Wolong	Gengda	Total (%)
	Samples	61	76	137 (100)	81	101	182 (100)
Gender	Female	24	35	59 (43.1)	46	52	98 (53.8)
	Male	37	41	78 (56.9)	35	49	84 (46.2)
Age	<30	21	18	39 (28.5)	10	12	22 (12.1)
	30–50	23	39	62 (45.2)	51	69	120 (65.9)
	>50	17	19	36 (26.3)	20	20	40 (22.0)
Education level	≤Primary	27	43	70 (51.1)	30	46	76 (41.8)
	Middle school	25	21	46 (33.3)	26	29	55 (30.2)
	≥High school	9	12	21 (15.3)	25	26	51 (28.0)

### 3.2 Changes in natural capital

**Figure 1:**
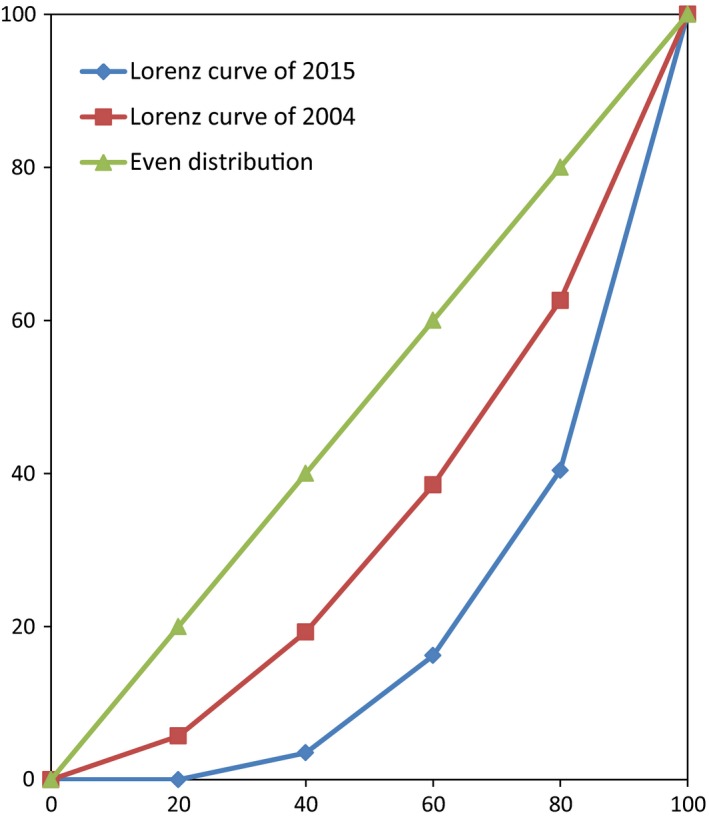
Lorenz Curve of respondents’ cropland holding in household

From 2004 to 2015, the average area of cropland per household and the per capital cropland area decreased by approximately 31% and 15%, respectively (Table 2). The area of reforested land per household increased by approximately 17.51%. The distribution of cropland among respondents in 2015 had become more uneven than in 2004 (Figure 1). Approximately 28% of respondents had lost all of their cropland in recent years, and in 2015, 60% of the cropland area was owned by approximately 20% of respondents.

**Table 2:** Changes in local natural capital

Indicators	2004	2015	Growth (ha)	Growth rate (%)
Average cropland area per household (ha)	0.223	0.153	−0.07	−31.39
Per capital cropland area (ha)	0.040	0.034	−0.006	−15.00
Average reforested land area per household (ha)	0.297	0.349	+0.052	17.51

### 3.3 Changes in financial capital

There were dramatic changes in household financial capital in recent years. Table 3 shows that both average total income per household and per capita income increased greatly, by 433% and 511%, respectively. Although average agricultural income also increased substantially (i.e., by 245%), its proportion of total household income decreased by approximately 35%. In contrast, local nonagricultural income increased by 805% and its proportion of total household income increased to 70%.

**Table 3:** Changes in financial capital

Indicators	2004	2015	Growth	Growth rate (%)
Average total income per household (Yuan)	5,913.8	31,488.4	25,574.6	432.5
Per capita total income (Yuan)	1,182.8	7,228.9	6,046.1	511.2
Average agricultural income per household (Yuan)	2,266.7	7,824.4	5,557.7	245.2
Proportion of agricultural income to total income (%)	38.3	24.9	−13.4	−35.0
Average nonagricultural income per household (Yuan)	2,468.8	22,359.2	19,890.4	805.7
Proportion of nonagricultural income to total income (%)	41.75	71.01	29.26	70.0
Average compensation (cash + grain) per household (Yuan)	1,759.1	970.1	−825	−46.9
Per capita compensation (cash + grain) (Yuan)	337.85	223.1	−114.75	−34.0
Proportion of compensation to total income (%)	29.75	3.1	−26.65	−89.9
Proportion of compensation to agricultural income (%)	77.6	12.4	−65.2	−84.0
P90/p10 of total income per household	6.7	7.7	1.0	16.8
P90/p10 of agricultural income per household	6.4	20.0	13.6	212.5
P90/p10 of nonagricultural income per household	30.8	19.0	−11.8	−38.3

In the first stage of the GGP, cash and grain were provided to the local people as compensation for their cropland conversion, while in the second stage only cash was provided as compensation to local people. For this study, the grain compensation (i.e., 2,250 kg/ha) was converted to a cash value according to the grain price in 2003 (i.e., 2.5 Yuan (RMB)/kg). Hence, the 2004 “cash + grain” values in Table 3 represent a combined cash value for comparative analysis with the 2015 cash values. Compared to other income, the average compensation provided to local households from the GGP reduced significantly between 2004 and 2015 (by approximately 47%), and its proportion of total household income and agricultural income both decreased even more so, by 89.9% and 84.0%, respectively.

There were varying changes in the distribution of income among households. Average agricultural income per household became more unevenly spread, with the p90/p10 increasing from 6.4 to 20.0 (an increase of 212.5%). Average nonagricultural income per household became more evenly spread, with the p90/p10 decreasing by approximately 38%. Average total income per household also became more unevenly spread.

### 3.4 Changes in human capital

Changes in household human and material capitals are outlined in Table 4. Human capital comprised two indicators: the number and ages of people per household, and their level of education. The level of education among respondents increased substantially between 2004 and 2015. In particular, the number of respondents that had attained a high school education increased by 83%. Respondents and their family members were classified according to their age to discern the potential local labor force. Compared to 2004, the number of people aged under 15 had decreased by approximately 55% in 2015 and the number of people aged between 16 and 20 had decreased by approximately 40%. It could therefore be deduced that the potential local labor force will be reduced in the future years. The potential adult labor force (i.e., household members aged between 15 and 50) increased to varying degrees, with the fastest growth in potential workers in the 41–50 years age bracket. In terms of the numbers of people in the potential labor force, most were aged between 21 and 30 followed by those aged between 31 and 40. These two age groups of the potential labor force comprised 42% of the total respondents and became the largest stakeholders for off‐farm employment.

**Table 4:** Changes in human capitals

Indicators	2004	2015	Growth	Growth rate (%)
Proportion of people aged under 15	25.5	11.3	−14.2	−55.7
Proportion of people aged between 16 and 20	11.8	7.1	−4.7	−39.8
Proportion of people aged between 21 and 30	19.2	23.4	4.2	21.9
Proportion of people aged between 31 and 40	16.7	19.0	2.3	13.8
Proportion of people aged between 41 and 50	8.3	16.4	8.1	97.6
Proportion of people aged between 51 and 60	8.2	10.0	1.8	22.0
Proportion of people aged >60	10.3	12.9	2.6	25.2
Proportion of people ≤ primary education level	51.1	41.8	−9.3	−18.2
Proportion of people with middle school education level	33.6	30.2	−3.4	−10.1
Proportion of people ≥ high school education level	15.3	28.0	12.7	83.0

### 3.5 Changes in material capital

Changes in household material capitals are outlined in Table 5. For the household material capital, the number of households rearing pigs and cattle changed little, but there were greater changes in the proportions of total households rearing pigs and cattle, equating to decreases of approximately 31% and 22%, respectively, since 2004. A deeper conversation with respondents found that local people raised cattle mainly for agricultural assistance in 2004 but for meat sales in 2015. The average number of cattle per household increased by approximately 158%. In addition, mass breeding had appeared by 2015, with, for example, two households raising more than 35 pigs for commercial sales in that year.

**Table 5:** Changes in material capitals

Indicators	2004	2015	Growth	Growth rate (%)
Number of households feeding pigs	108	100	−8	−7.4
Proportion of total households feeding pigs (%)	78.8	54.6	−24.2	−30.8
Average number of pigs per household	3.86	3.55	−0.31	−8.0
Number of households feeding cattle	32	33		3.1
Proportion of total households feeding cattle (%)	23.3	18.1	−5.2	−22.3
Average number of cattle per household	4.06	10.5	+6.44	158.6

### 3.6 Changes in livelihood strategy

The GGP aimed to reduce farmers’ dependence on agriculture and encourage redundant rural labors to find other nonagricultural employment. We defined the labor force as those household members aged 18 or older. Every family member of the surveyed households was asked about his/her occupation and so we were able to determine the number of labor force that was employed in nonagricultural industries. The results showed that nonagricultural employment increased substantially since 2004 (Table 6). The proportion of the local population and the potential local labor force employed in nonagricultural industries increased by 250% and 153%, respectively. The local workers were categorized as either regular employees or temporary employees. Regular employees had stable employment with good social welfare such as hospital cover and social security. Temporary employees had unstable employment with associated high mobility and uncertainty, and limited social security. Ordinarily, temporary employees retain a strong connection to agriculture and would be likely to return to rural areas and agricultural employment if they lost their jobs or when they were older. Our results showed that more respondents were engaged in temporary employment than regular employment. The proportion of temporary employees in the local population and the potential local labor force were approximately 27% and 61%, respectively. This was higher than the respective percentages of regular employees (i.e., approximately 9% and 19%, respectively).

**Table 6:** Changes in livelihood strategy

Indicators	2004	2015	Growth	Growth rate (%)
Proportion of the local population employed in nonagricultural industries	10.2	35.7	25.3	250.1
Proportion of the potential local labor force employed in nonagricultural industries	31.5	79.9	48.8	153.7
Proportion of the local population in regular nonagricultural employment	2.8	8.6	5.8	205.7
Proportion of the potential local labor force in regular nonagricultural employment	8.7	19.2	10.5	121.4
Proportion of the local population in temporary nonagricultural employment	7.4	27.2	19.8	266. 9
Proportion of potential local labor force in temporary nonagricultural employment	22.8	60.7	37. 9	166.0

## 4 DISCUSSION

There have been dramatic changes in livelihood capitals and distribution among households in our study area over the past 10 years. In general, it is optimistic to have found a dramatic decrease in local cropland holdings and a dramatic increase in the households’ nonagricultural income, as these outcomes indicate that the locals’ dependence on agriculture has decreased and that redundant rural workers have shifted away from growing crops. These changes align with the initial objective of the GGP. Although the GGP is not the only driver of these changes, it is now confronted with new socio‐economic conditions in our study site and will have to be adjusted if its planned next stage is to be an effective and smooth implementation. We highlight the following three challenges for the sustainability of the GGP's planned next stage in our study site.

### 4.1 Challenge 1: Heterogeneity in agricultural dependence

There were distinct uneven distributions of cropland holdings and agricultural income among the respondents. At least two typical groups were evident among the respondents—landless respondents and land‐secure respondents. In the study area, it has been established that households with more cropland are less willing to re‐enroll in the GGP due to their dependence on farming (Xu, Kong, Liu, & Wang, 2017). Thus, uneven distribution of cropland holdings deserves consideration in next stage of the program. Yin, Liu, Yao, and Zhao (2013) have suggested a need to target disadvantaged households and communities because they are more likely to reconvert some of their afforested cropland back to farming once the program expires. Liu et al. (2010) also remind us that certain households with limited engagement in off‐farm activities and that have continued their dependence on farming for their livelihood are significant stakeholders in the GGP. These two significant groups bring about distinct issues for the next stage of the program. For the landless respondents, most have had to transfer to off‐farm industries due to their lack of access to cropland. For the land‐secure respondents, their high dependence on cropland could impede their re‐enrolling in the next stage of the program. Therefore, we advise GGP designers and managers to take local household heterogeneity into consideration and implement targeted measures aimed at encouraging local re‐enrollments in the next stage of the GGP.

### 4.2 Challenge 2: Decreasing ecological compensation and increasing value of cropland

Compared to the rapid growth in household agricultural and nonagricultural income between 2004 and 2015 (245.2% and 805.7%, respectively), compensation paid to households for their cropland conversion decreased substantially (−46.9%). The proportion of this ecological compensation to household total income and agricultural income decreased even more so (−89.9% and −84.0%, respectively). This is a result of the continued decline in compensation from the first stage to the third stage of the GGP (http://tghl.forestry.gov.cn/portal/tghl/s/2166/content-846817.html) and the obvious increase in local incomes. In the first stage of the program, the absolute value of compensation was lower than cropland income, because most local croplands were under cabbage cultivation (Xu et al., 2007). At present, the compensation for converting cropland to forest remains far below the actual cropland revenue potential.

In contrast to the rapid decline in ecological compensation, the value or cost of local cropland was increasing because of the emergence of agricultural cooperatives which lease local cropland for commercial, market‐oriented cultivation. The amount these organizations pay to lease the cropland is higher than the GGP compensation (Xu et al., 2017). It was also evident that some respondents were changing their livelihood strategy from subsistence farming to market‐driven farming. Pig and cattle breeding, which in the past served as a means of supplying meant for family consumption and draft power, has increasingly turned to a focus on commercial sales. In addition, there have been obvious rises in the costs of food, houses, and other living‐related items in China over recent years (Guo, Li, Yu, & Hao, 2011).

As market‐based instruments, PES programs should at least cover the opportunity costs of the changes to land management necessary for suppliers to provide the sought‐after environmental services. Thus, decreasing compensation, together with the increasing costs of cropland and living items, will challenge the next stage of the GGP. The fact that decreasing compensation from the GGP might induce participants to abandon the program and return to farming has been a concern expressed by other researchers (Cao, Chen, Chen, & Gao, 2007; Zhen et al., 2014). In the study area, croplands were converted to ecological forests that aside from the ecological compensation are providing no direct productive value (such as from wood or fruit) for the local people. Hence, the emergence of market‐driven farming has provided the local households with a better understanding of the opportunity cost of cropland conversion and how this compares with the ecological compensation they could receive from the GGP. This ecological compensation needs to be adjusted to better reflect local expectations.

### 4.3 Challenge 3: Pressures on labor supply

The long‐term success of a PES program depends on whether a local labor force can find a livelihood alternative to growing crops. In the study area, a positive finding was that the level of education among respondents had substantially increased. This may be conducive with their re‐enrolling in the next stage of the GGP (Xu et al., 2017). Relating the observed changes in human capital and livelihood strategies to the next stage of the program, we found two key challenges for the program: One is that the potential adult labor force aged between 21 and 40 has increased and is presently at a peak; the other is that most off‐farm workers are engaged in temporary, high‐mobility nonagricultural jobs that have little technical requirements and social security such as the provision of a pension. This meant that these off‐farm workers were not completely divorced from agriculture. As we found in the survey, most temporary employees stated that they would return to work on the farm if they lost their job or when they were older. Together, the high pressure on the local labor force plus the high potential for nonagricultural employees to return to the farm will challenge the next stage of the GGP.

The GGP itself actually has a small positive effect on nonfarm employment (Kelly & Huo, 2013). The program should not be limited to stipulations themselves, but should resort to other related policy measures (Yin, Xu, Li, & Liu, 2005). This is important because all policies concerning rural land and the labor market can alter the opportunity cost of land‐use and eventually influence the incentive for farmers to participate in a cropland conversion program and the likelihood that they will convert their afforested land back to cropping (Yao, Guo, & Huo, 2010). Yin et al. (2013) have also emphasized the need to explore the interactions of different policies to achieve program effectiveness. Here, we find some opportunities for a transformation of the local labor force to nonfarming industries. Firstly, younger workers are more competitive to accept new technology and information, thereby strengthening their capabilities in an increasingly competitive employment market. Secondly, Wolong National Nature Reserve is promoting the development of local tourism resources and an increasing number of local households are becoming involved in the tourism industry in the form of farmhouse resorts (also named “Nongjiale” in Chinese). Local people have high expectations for local tourism development and hope that this will result in improved local employment opportunities (Xu et al., 2017). Finally, with Sichuan Province being the largest labor export market in China, the provincial government is now working to attract its exported labor force back to their home to start new businesses (http://www.sc.gov.cn/10462/12771/2017/2/21/10414645.shtml).

## 5 CONCLUSIONS

It is well‐known that PES or restoration programs should be adaptable to changing socio‐economic and environmental conditions at their target locations. In this context, our research contributes a case study of the household livelihood changes associated with implementation of the GGP in the Wolong National Nature Reserve in southwestern China and the implications of these changes for the sustainability of the program. A number of conclusions are made:

1. The livelihood changes expected by the program were not evenly distributed among the participants. This has varying effects for households and PES program designers should therefore regularly review such contextual changes and adjust the program accordingly. A bottom‐up approach and the precise targeting of disadvantaged households are recommended when reviewing the contextual changes;
2. As an economic and market‐oriented method, the costs and benefits of a PES program change with the general socio‐economic context. Competitive economic incentives are needed to sustain the program and ensure ongoing provision of ecosystem services; and
3. PES programs cannot be divorced from other policies. Changes in participant characteristics and the effects of these changes are out of the control of the program itself. Hence, coordinating with other related land‐use policies is advised to advance a PES program.

As both society and the environment are not static, a constant PES will lag behind the socio‐economic and environmental context at its targeted locations. Ensuring a PES is dynamic according to it targeted context will contribute to its long‐term success. Taking both household average and unevenly distributed changes associated with participation in a PES program into consideration in a holistic way provides new insights for PES implementation. Contextual changes happen for individual households, the broader community, and at the district/national level. Household‐level analysis is an important means of understanding the fine‐scale contextual changes and their implications for the design and implementation of targeted PES programs. In the long term, PES program should establish a regular monitor system for typical households in the community and inform the significant feedback of changes to program managers.

## CONFLICT OF INTEREST

The authors declare no conflict of interest.

## AUTHOR CONTRIBUTIONS

Jianying XU designed the research, conducted field work in 2004 and 2015, and wrote the manuscript. Qing WANG and Ming KONG conducted field work in 2015.
